# A survival analysis of dropout among French swimmers

**DOI:** 10.3389/fspor.2025.1509306

**Published:** 2025-03-05

**Authors:** Audrey Difernand, Alexia Mallet, Quentin De Larochelambert, Robin Pla, Andy Marc, Kilian Barlier, Juliana Antero, Jean-François Toussaint, Adrien Sedeaud

**Affiliations:** ^1^ IRMES—URP 7329, Institut de Recherche Médicale et d’Epidémiologie du Sport, Université Paris Cité, Paris, France; ^2^ Pôle Performance, INSEP, Institut National du Sport, de l'Expertise et de la Performance, Paris, France; ^3^ Département Optimisation de la Performance, Fédération Française de Natation, Clichy, France; ^4^ Centre d’Investigation en Médecine du Sport, Assistance Publique—Hôpitaux de Paris, Hôtel-Dieu, Paris, France

**Keywords:** dropout, swimming, youth sports, talent identification, Kaplan–Meier analysis, athlete retention

## Abstract

This study examines the dropout rates among French swimmers based on performance levels, sex, and relative age. Using data from 160,861 swimmers under the age of 21, we analyzed the distribution of birth quarters and dropout rates across performance levels. Chi-squared tests were conducted to confirm the significant effect of birth quarter on performance. Kaplan–Meier Survival (KMS) curves were used to evaluate and interpret the impact of sex and relative age on dropout trends. The results show that dropout peaks occur at 13.16 years for girls and 17.50 years for boys. Analyzing by age year, at 13 years, the top 10% of female swimmers exhibit a dropout rate of 8.7% (9.9% for males), while the bottom 10% show a much higher rate of 78.1% (69.3% for males). By 17 years, the dropout rate rises to 39.6% (28.6% for males) for the top 10% and 91.7% (83.4% for males) for the bottom 10%. KMS curves, stratified by age, reveal similar dropout trends for both sexes below the age of 13. However, after this age, the dropout rate increases more sharply among females, reaching a maximum difference of 4.8% at 17.9 years. Disparities in dropout rates based on birth quarters are most pronounced at 12.7 years for girls (10%) and at 14.7 years for boys (8.1%). This study underscores the significant influence of sex, relative age, and performance level on dropout rates among French swimmers. Higher performance levels are associated with lower dropout rates, and female swimmers display consistently higher dropout rates than their male counterparts.

## Introduction

Dropout in youth sports is defined as the termination of participation in a specific sport, without necessarily ceasing participation in all sports (1). It represents a critical challenge in the development of young athletes, with wide-reaching implications for both the individual and the broader sports ecosystem. Dropout is often linked to factors such as loss of interest, boredom, attraction to other activities, conflicts with coaches, injury frequency, stagnation in performance, and a lack of enjoyment (2, 3). Conversely, sustained participation is frequently associated with positive parental support, intrinsic satisfaction from the sport, and rewards derived from success (3). However, excessive pressure during adolescence can lead to disengagement (4), further complicating the retention dynamics in youth sports. While these general factors have been widely documented, the interplay of sex, performance level, and the Relative Age Effect (RAE) in explaining dropout patterns remains underexplored, particularly in the context of competitive swimming.

The concept of RAE has been widely studied in team sports, revealing an overrepresentation of athletes born early in the selection year due to developmental advantages (1). However, its impact on individual sports like swimming remains underexplored, particularly in the context of dropout. This study addresses this gap by analyzing dropout rates among French swimmers with respect to sex, performance level, and birth quarters. Investigating these factors is crucial, as dropout trends differ significantly between boys and girls, often peaking during puberty (5), and are influenced by performance pressures and relative age disadvantages.

The present study is grounded in three key hypotheses to address this gap. First, we hypothesize that dropout rates will differ between male and female swimmers, with female athletes experiencing higher dropout rates at an earlier age. This hypothesis is supported by prior studies highlighting gender disparities in youth sports participation, as well as the unique pressures often faced by female athletes during adolescence. Second, we anticipate that RAE, a phenomenon in which athletes born earlier in the selection year gain a relative advantage in physical and cognitive maturity compared to their peers born later, will play a role in dropout patterns. Specifically, swimmers born in the first quarters of the year are expected to have lower dropout rates than those born in later quarters, as the advantages conferred by RAE have been shown to impact both performance and selection in youth sports. Finally, performance level is hypothesized to significantly influence dropout rates, with higher-performing swimmers—those in the top deciles of performance—being more likely to persist compared to lower-performing swimmers. This aligns with previous findings suggesting that success in competition fosters continued engagement, creating a virtuous cycle of commitment and achievement.

While several studies have examined dropout rates in youth sports, most rely on descriptive statistics rather than more robust methods such as survival analyses. Kaplan–Meier Survival (KMS) analyses and Cox regression models, traditionally used in medical research, offer a powerful framework for investigating dropout rates. These methods account for censored data, enabling a more nuanced understanding of persistence over time. For example, in a study of Flemish gymnasts, only 17.6% of athletes remained in the high-performance pathway for more than five years, with an average survival time of 2.5 years (6). Similarly, survival analyses applied to Canadian female soccer players revealed that only 23.3% persisted over a seven-year period, with disparities in survival times based on birth quarters (7). Such findings underscore the utility of survival analyses in identifying dropout patterns across various sports.

The concept of RAE adds another layer of complexity to youth sports retention. RAE, which reflects the advantage of being relatively older within an age group, has been shown to influence performance and participation in a wide range of sports. For instance, Canadian female soccer players born in the first quarter of the year demonstrated longer survival times in the sport compared to those born later in the year (7). However, the extent to which RAE influences dropout varies by sport and context. In swimming, RAE has been shown to impact young French swimmers regardless of sex or competitive level (8). This suggests that while RAE is a relevant factor, its interaction with other variables, such as performance level and sex, warrants further investigation.

Swimming provides a unique context to study dropout and RAE because it is both highly competitive and performance-oriented, with objective and standardized metrics for success. Prior research among Australian swimmers identified factors such as age, level of competition, and proximity to urban centers as significant predictors of dropout, whereas sex and relative age appeared less influential (1). However, no study to date has examined dropout in French swimmers using an integrative approach that simultaneously considers sex, RAE, and performance level. The present study seeks to fill this gap by investigating dropout patterns among French male and female swimmers, with a particular focus on the 50 m freestyle event—the most widely contested swimming event. By examining the interplay of sex, performance levels, and birth quarters, this study aims to provide novel insights into the mechanisms underlying dropout in swimming. Understanding these dynamics is essential for identifying at-risk athletes and developing targeted interventions for coaches, policymakers, and sports federations to foster long-term engagement in the sport.

## Materials & methods

### Data collection

The database from the French Swimming Federation provides all French participation in all types of competitions (local to international) from 2002–2020 in all individual events (50 m, 100 m, 200 m, 400 m, 800 m, 1,500 m Freestyle, 50 m, 100 m, 200 m Breaststroke, Backstroke and Butterfly). For each swimmer, birthdate, performance time and competition are available. All performances took place in 50-meter pools, which is the official Olympic standard length. Only swimmers who started competition before the age of 18 and competed at least two seasons were considered. Swimmers (50.8% females) were divided into age categories. According to the French Swimming Federation, the female age categories are as follows: Kids: under 10 years old, Youth: between 11 and 13 years old, Junior: between 14 and 16 years old, Senior: over 17 years old. For males, the age categories are shifted up by one year. At each age, swimmers are divided into performance decile, according to their best performance. A performance decile is a method of dividing a ranked dataset into ten equal parts, with Decile 1 representing the top 10% of swimmers (highest performance) and Decile 10 the bottom 10% (lowest performance). Indeed, for our analysis Decile 1 includes the top 10% of swimmers (highest performance), Decile 2 represents the 10%–20% performance range, Decile 3 covers the 20%–30% range, and so on, ending with Decile 10, which includes the bottom 10% of swimmers (lowest performance). This classification provides a consistent and comparable metric for assessing swimmers' relative performance each year, independent of competition levels or age groups. By using deciles, we can intuitively analyze trends, such as how dropout rates vary across performance levels, allowing us to compare top-performing and bottom-performing swimmers without being limited to specific events or competitions.

### Relative age effect

The distribution of the birth quarters (Q1: January–March, Q2: April–June, Q3: July–September, Q4: October–December) was calculated for each decile. Chi-square tests was run for each age category to identify significant differences of birth quarters between performance deciles.

### Dropout

Dropout was defined as swimmers disappearing from the database before age 21 or being absent for at least two consecutive seasons before 21. The starting age was the swimmer's first recorded competition. Dropout rates were analyzed by performance decile, event, and sex, using linear regression to test the relationship between decile and dropout rate. The analysis ended in 2020 to avoid the confounding effects of the COVID-19 pandemic, which disrupted sports participation. A two-year absence threshold was chosen to account for temporary absences, such as injuries, ensuring that only permanent dropout was identified. Swimmers competing continuously until 21 or with interruptions of less than two years were not classified as dropouts. We measure the dropout proportion for each performance decile, event and sex. The dropout rate “*r*” at age “*t*” will be expressed as a function of the membership of performance decile *D* at age *t*-1. At each age, we perform a linear regression between the dropout rate and decile:

r=a*D+b

We perform a Student test to test the hypothesis that the coefficient “*a*”, representing the additional percentage per decile, is different from 0 at threshold 0.05.

### Survival analysis

To study the evolution of the onset of dropout according to sex and birth quarter, KMS curves were estimated. This method was particularly adopted here as it allows to deal with swimmers who started swimming at different times in their life, considering starting age. In this study, KMS curves were plotted according to relative age in order to compare athletes at a similar stage of their career. Kaplan Meier estimates were computed to determine differences in terms of dropout between birth quarter for each sex. To assess the significance of the results, log-rank tests were performed for each considered groups. This test checks whether KMS curves are significantly different from each other.

## Results

160 861 athletes who started swimming before the age of 18 are included in this study (49.2% male). Among those who are at least 21 years old and have been practicing for at least two seasons, 79.1% of male and 87.2% of female have dropped out of swimming. Regardless of the age at which the performances are carried out, girls drop out the most at 13 and boys at 17. When we look at the ratio of these frequencies to the population of the age concerned, the dropout rate is higher at 17 for both girls and boys.

**Figure 1:**
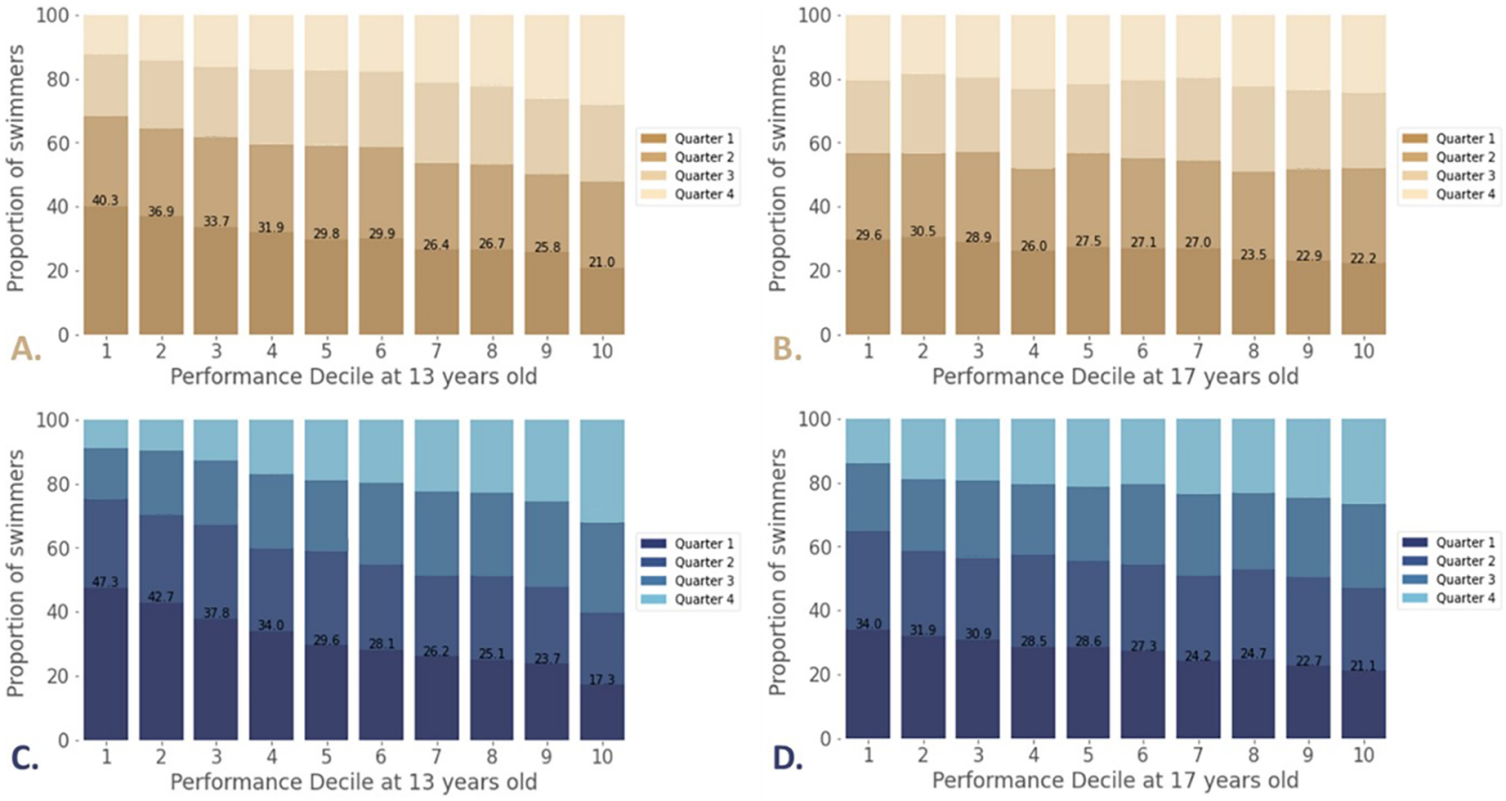
Distribution by birth quarter and performance decile of swimmers at 13 **(A)** and 17 **(B)** years old in the female 50 m freestyle and of swimmers at 13 **(C)** and 17 **(D)** years old in the male 50 m freestyle.

The distribution of swimmers by birth quarters within each performance decile is shown in Figure 1 for the 50 m Freestyle male and female event in the 50 m pool at 13 and 17 years old. The birth quarter distribution between Q1 and Q4 for all other events is shown in Supplementary Table S1 in Supplementary File. Swimmers with at least one performance time at 13 and who are more than 15 years old at the last recorded competition are considered here (similarly for 17 years old swimmers, only those with at least one performance time who are older than 19 are included). By conducting a chi-square significance test on all events, females support a significant effect from birth quarter on performance decile from 13–18 years of age (and from 13–19 years of age for males, except in breaststroke; *p*-value < 0.05).

At age 13 in decile 1, 40.3% of female swimmers are born in Q1 vs. 12.4% in Q4 (Figure 1A), while 47.3% of males are born in Q1 vs. 8.7% in Q4, (Figure 1C). The proportion of Q1 decreases significantly and the proportion of Q4 increases significantly as performance level decreases, from decile 1 to decile 10.

At age 17, the difference is smaller but still present: 29.6% of females belonging to decile 1 are born in Q1 vs. 20.5% in Q4, (Figure 1B) while 34.0% of males are born in Q1 vs. 14.1% in Q4 (Figure 1D).

**Figure 2:**
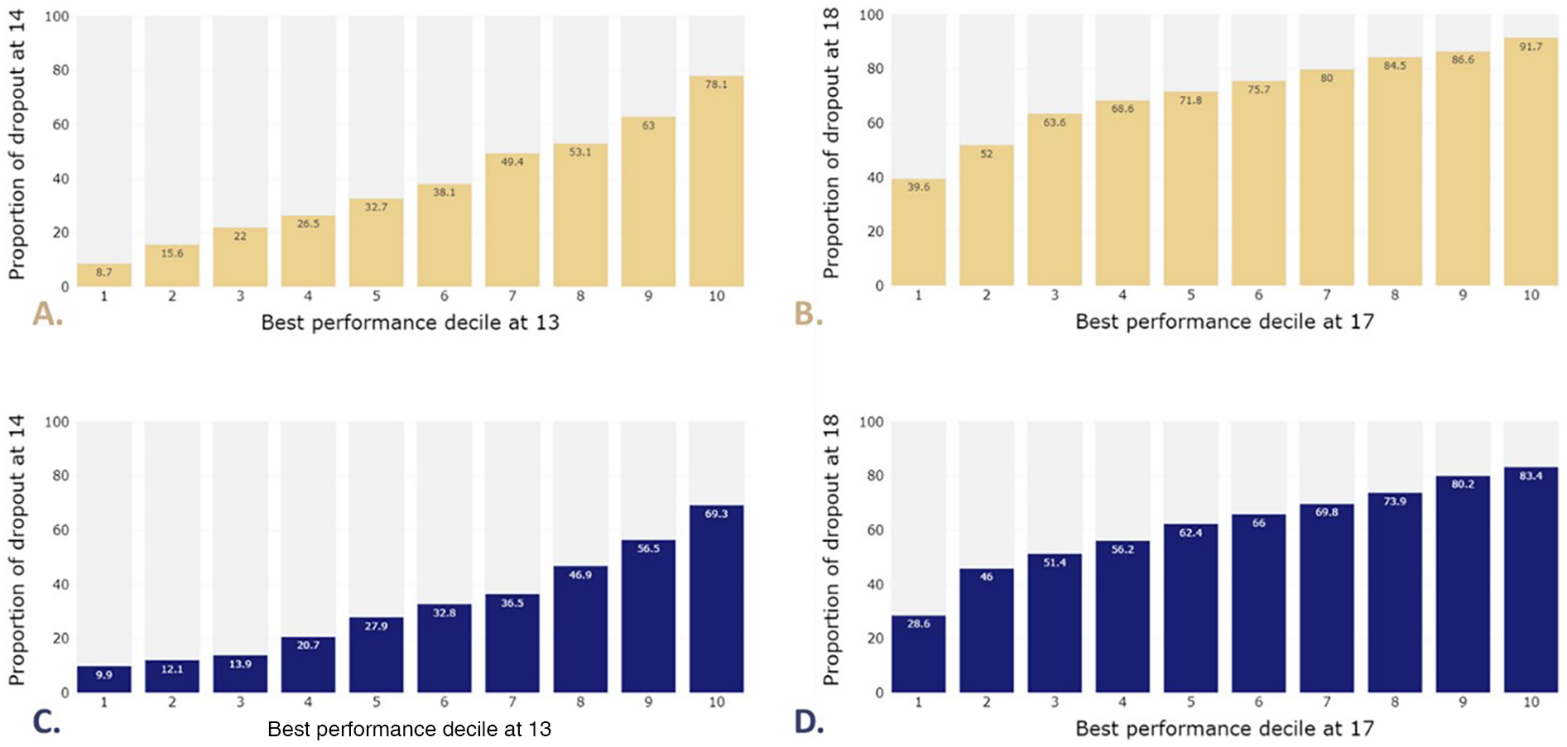
Dropout rate at age 14 based on performance deciles at age 13 in the female **(A)** and male **(C)** 50 m freestyle in the 50 m Olympic swimming pool. Dropout rate at age 18 based on performance deciles at age 17 in the female **(B)** and male **(D)** 50 m Freestyle in the Olympic swimming pool (50 m).

The evolution of the dropout rate by performance decile for the female and male 50 m Freestyle is presented in Figure 2. Chi-squared tests highlight the significant effect of the performance decile at age *t* on dropout at *t* + 1 in both sexes from age 8 onwards. The dropout rate increases significantly with performance decile. The dropout rates at ages 14 and 18 according to performance deciles 1 and 10 at ages 13 and 17 respectively are presented in Supplementary Table S2 in Supplementary File.

**Figure 3:**
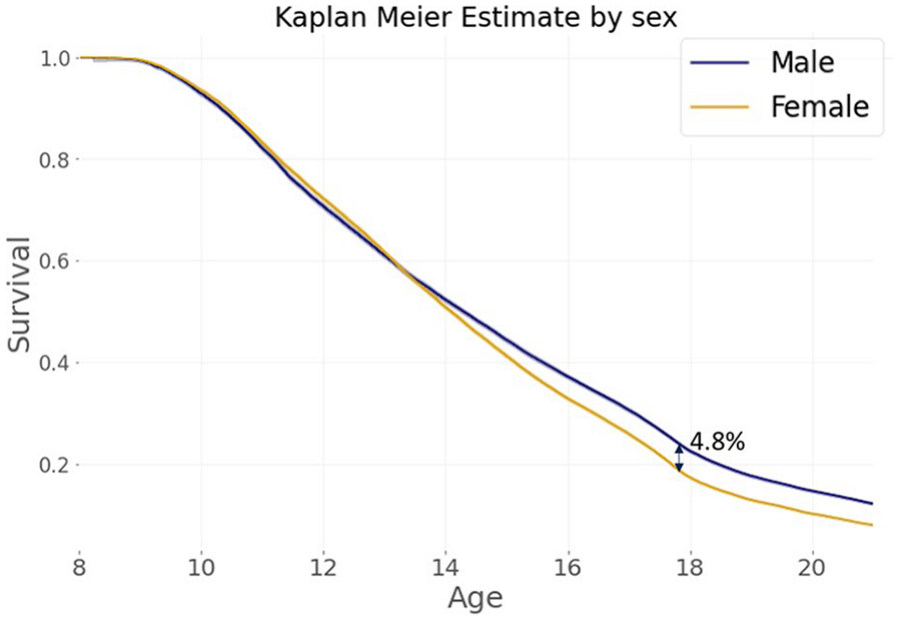
KMS curves by sex.

The KMS curves according to sex are presented in Figure 3. 74,922 males and 80,170 females started swimming at 8 years or later. The KMS curves are similar before the age of 14; then, the difference between the two curves becomes significant from the age of 15 onwards (log-rank test: *p*-value < 0.005): females drop out significantly more than males. The greatest difference between both sexes occurs at 17.89 years, where females are 4.8% more likely to drop out than males.

**Figure 4:**
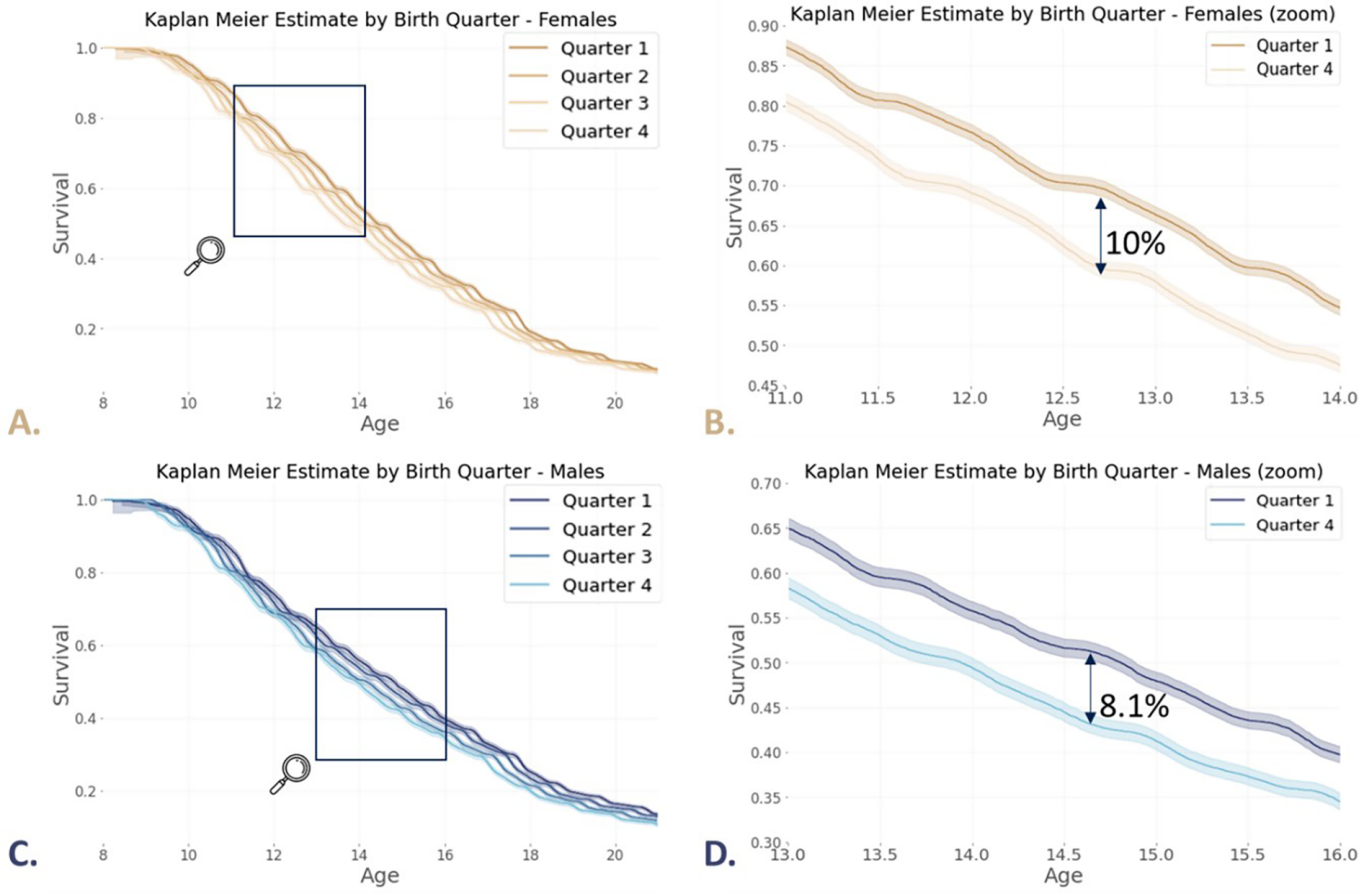
**(A)** KMS curves according to birth quarters for females **(B)** zoom in on the first and last quarter KMS for females between 11 and 14 years of age **(C)** KMS curves according to birth quarters for males **(D)** zoom in on the first and last quarter KMS for males between 13 and 16 years of age.

The KMS curve for quarter 1 is always above that of the other quarters from age 10 onwards for females (log-rank test: *p*-value < 0.005, Figure 4A) and from age 13 onwards for males (log-rank test: *p*-value < 0.005, Figure 4C), while the KMS curve for quarter 4 is always below that of the other quarters. Focusing only on the difference between the quarter 1 and quarter 4, the largest observed difference between the two corresponding KMS curves occurs at 12.71 years old with a 10% difference (Figure 4B) and at 14.67 years old with 8.1% (Figure 4D).

## Discussion

This study highlights the impact of sex, birth quarter and performance level on dropout rates for all swimming events among 8–21 years old swimmers. It shows that the dropout rate increases when the level of performance decreases. The Kaplan Meier survival curves show a similar evolution of dropout rates for girls and boys until the age of 13, after which girls tend to drop out significantly more than boys, who face a peak in dropout around the age of 17.

### Dropout and birth quarter

A relative age effect is found within the performance deciles, particularly at age 13, for both girls and boys, in accordance with previous study (9). In the top performance decile, the proportion of swimmers born in the first quarter of the year is consistently higher than the proportion born in the last quarter (3). Taking the top 10% of a sample of Australian swimmers (equivalent to our performance decile 1), at ages 13, 14, 15 and 16, the proportion born in Q1 is 56%, 42.9%, 50% and 36.4%, while that born in Q4 is 4%, 8.9%, 8.3% and 14.5% respectively. At 13, 14 and 15, the authors identify a large effect size and a medium one at 16 years of age. Difernand et al. (9) also highlight a relative age effect accentuated by the level of performance in French 50 m Freestyle swimmers. In a study (4), reveal a similar trend in 12–15-year-old 100 m and 200 m breaststrokers, with more Q1 born swimmers in the top 10% than Q4.

Yet, month of birth is not the only birth-related variable that affects dropout. The place of birth also seems to have an influence (6). Brazilian footballers, who were born in the first half of the year in a city with less than 100,000 inhabitants and a human development index (dimensionless composite index between 0 and 1, calculated on the basis of the quality of life of their citizens: health, education, standard of living) above 0.501 are more likely to play in the top teams of the Brazilian football league compared to those whose born in the second semester (6). Good access to health and education facilities may encourage sports participation, especially among young people (6), and human development index may favor the development of sport (7).

### Dropout and performance

We find that the dropout rate is lower when performance level increases. It may be due to the coaches paying more attention to the best swimmers of the category, in the quest of future medals. The relationship with the coach and manager and the attention given to young athletes is a determining factor in future success (8). To our knowledge, there is no study yet that has quantified dropout by distinguishing performance levels. However, Rottensteiner (10) and Eystein mentioned stagnation or decline in performance as a major cause of low self-esteem, lack of motivation and dropout (11). Attempting to achieve a higher performance may turn into an obsession and eventually becomes a source of demotivation (11). Lack of success is one of the major factors for withdrawal from sport (12). In addition, the desire to spend more time with friends or practice other activities, with time spent away from sport or school is important (10, 11, 13). In particular, some “dropped-out” swimmers considered competitive swimming as less important than other activities (12), with a high level of burnout within competitive swimming (14). When the return on investment is not as good as hoped for, and the results not as expected, the heavy volume of training and the relationship with the coach tend to be blamed (13). Indeed, swimmers find training intolerable when they are not satisfied with the results.

### Dropout age and sex

Our sample of females swimmers present a dropout rate significantly higher than their males' counterparts (87.2% vs. 79.8%), in accordance with previous studies (15). Among the population of French swimmers, females drop out the most at 13 years of age and males at 17, a trend similar to young French footballers (16, 17). This highest probability of dropping out of swimming at age 13 for girls is consistent with the difference in the two survival curves between the first and last quarters, which peaks at 12.71 years. Erlandson et al. (18) investigated growth and maturation in gymnastics, swimming and tennis among 222 athletes, and suggested that puberty could be an explanatory factor. With the menarche, the body changes which may affect negatively the motivation and slow down the sport practice, with the acceptance of a new morphology, the management of menstruations, the appearance of body hair while going to the pool or the difficulty to fit in a swimming suit.

For boys, on the other hand, the maximum dropout difference between the first quarter and the last quarter occurs at 14.61 years of age, almost two years later than for girls. Once again, the connection with the onset of puberty may be questioned. Peak of Height Velocity (PHV), *i.e.,* the time of maximum height growth, generally occurs later in boys (between 13 and 15 years) than in girls (between 11 and 13 years) (19, 20). Also, the fact that the probability of boys dropping out is highest at 17 may be linked to the moment of moving out from high school and therefore to a choice to be made. A study (21) of 169 student athletes in team sports (basketball, baseball, softball, volleyball) and individual sports (tennis, golf, cross country, swimming) revealed that more than half of the students report that it is difficult to match both athletic and academic expectations and that it is not easy to find enough time to study during their sport season.

To our knowledge, the only study using Kaplan Meier survival curves to investigate the dropout of swimmers was conducted by Moulds et al. (22). Among 7,895 female and 6,545 male swimmers, they found that 33% and 35% respectively dropped out after 2 years, while the median time of participation before dropout was 4 years. In a second part of the study, the authors sought to incorporate the notion of competition and considered 758 female swimmers and 603 male swimmers who are involved in competition. Among them, respectively 28% and 35% dropped out after 2 years of practice. The authors revealed that dropout was not associated with sex and birth quarter, unlike in our study. Indeed, by the age of 21, we show that females have significantly dropped out more than males. Other variables are included in the Moulds et al. study, such as socio-economic categories of the swimmer's living area, age category, level of competition, and proximity to a major city. These variables, which are related to the social developmental environment of the young swimmer, are found to be significantly more important and are the strength of this study. Møllerløkken et al. (15), in a meta-analysis showed that girls tend to have a higher dropout rate (26.8%) than boys (21.4%). Vilhjalmsson et al. (23) analyzed the sports habits of more than 3,000 Icelandic 6th, 8th and 10th grade students. They also found a higher dropout rate for girls at 34% and for boys at 29%. Their study showed that girls are less likely to join a sports club and are less physically active. In this study, girls reported practicing physical activity with their mothers or older sisters, while boys preferred practicing sport with their friends.

### Strengths & limitations

In swimming, some strokes require a high level of technique and a longer learning time. Swimming a 50 m freestyle is different from swimming a 1,500 m freestyle at 13 years old. Also, the return on investment may be delayed on longer distances, which may explain a higher dropout rate with the event distance. Experience was not taken into account in this study: some swimmers may regularly compete when others compete only once a year, but this may also be linked to the performance level. Other variables, such as information related to the club, training, school or familial environment, may also be relevant and generate future research. To strengthen the manuscript's impact, we propose actionable recommendations for coaches and policymakers based on our findings. Coaches should implement tailored retention strategies for younger swimmers, focusing on critical dropout ages—around 13 for girls and 17 for boys. Strategies like psychological support, individualized training, and mentorship programs can help sustain motivation and engagement. Policymakers should address the Relative Age Effect (RAE) by restructuring age-group categories or offering development programs for late-born swimmers, fostering inclusivity and long-term retention. Future research should explore interventions to mitigate RAE, investigate psychological and social factors influencing dropout, and analyze how specialization timing or multi-event participation affects retention. These efforts would provide a more holistic understanding of dropout and inform targeted strategies for swimmer retention.

## Conclusion

This study highlights the different impacts of sex and performance level on the dropout rate of young French swimmers. Girls drop out more frequently and earlier than boys while dropouts increase when performance level decreases. In that sense, since being highly performing at a young age is not necessarily a factor for success at the senior elite level (24–26), coaches and federation members could use this argument to explain to young athletes that they should not drop out based solely on performance.

## Data Availability

The raw data supporting the conclusions of this article will be made available by the authors, without undue reservation.

## References

[1] Cobley S Baker J Wattie N McKenna J. Annual age-grouping and athlete development: a meta-analytical review of relative age effects in sport. Sports Med. (2009) 39(3):235–56. 10.2165/00007256-200939030-00005 19290678

[2] Difernand A De Larochelambert Q Pla R Barlier K Marc A Ferri S Corrective adjustment methods for relative age effects on French young swimmers’ performances. PLoS One. (2023) 18(4):e0283229. 10.1371/journal.pone.0283229 37093823 PMC10124878

[3] Cobley S Abbott S Eisenhuth J Salter J McGregor D Romann M. Removing relative age effects from youth swimming: the development and testing of corrective adjustment procedures. J Sci Med Sport. (2019) 22(6):735–40. 10.1016/j.jsams.2018.12.013 30665755

[4] Abbott S Moulds K Salter J Romann M Edwards L Cobley S. Testing the application of corrective adjustment procedures for removal of relative age effects in female youth swimming. J Sports Sci. (2020) 38(10):1077–84. 10.1080/02640414.2020.1741956 32202222

[5] Eime RM Young JA Harvey JT Charity MJ Payne WR. A systematic review of the psychological and social benefits of participation in sport for children and adolescents: informing development of a conceptual model of health through sport. Int J Behav Nutr Phys Act. (2013) 10(1):98. 10.1186/1479-5868-10-98 23945179 PMC3751802

[6] Teoldo I Cardoso F. Talent map: how demographic rate, human development index and birthdate can be decisive for the identification and development of soccer players in Brazil. Sci Med Football. (2021) 5(4):293–300. 10.1080/24733938.2020.1868559 35077299

[7] Côté J Macdonald DJ Baker J Abernethy B. When « where » is more important than « when »: birthplace and birthdate effects on the achievement of sporting expertise. J Sports Sci. (2006) 24(10):1065–73. 10.1080/02640410500432490 17115521

[8] Ohl F Fincoeur B Lentillon-Kaestner V Defrance J Brissonneau C. The socialization of young cyclists and the culture of doping. Int Rev Sociol Sport. (2015) 50(7):865–82. 10.1177/1012690213495534

[9] Difernand A De Larochelambert Q Pla R Barlier K Marc A Ferri S Corrective adjustment methods for relative age effects on French swimmers’ performances. PLoS One. (2023) 18(4):e0283229. 10.1371/journal.pone.0283229 37093823 PMC10124878

[10] Rottensteiner C Laakso L Pihlaja T Konttinen N. Personal reasons for withdrawal from team sports and the influence of significant others among youth athletes. Int J Sports Sci Coach. (2013) 8:19–32. 10.1260/1747-9541.8.1.19

[11] Enoksen DE. Drop-out Rate and Drop-out Reasons Among Promising Norwegian Track and Field Athletes (2011).

[12] Brown BA. Factors influencing the process of withdrawal by female adolescents from the role of competitive age group swimmer. Sociol Sport J. (1985) 2(2):111–29. 10.1123/ssj.2.2.111

[13] Larson HK McHugh TLF Young BW Rodgers WM. Pathways from youth to masters swimming: exploring long-term influences of youth swimming experiences. Psychol Sport Exerc. (2019) 41:12–20. 10.1016/j.psychsport.2018.11.007

[14] Raedeke TD Smith AL. Development and preliminary validation of an athlete burnout measure. J Sport Exerc Psychol. (2001) 23(4):281–306. 10.1123/jsep.23.4.281 28682196

[15] Møllerløkken NE Lorås H Pedersen AV. A systematic review and meta-analysis of dropout rates in youth soccer. Percept Mot Skills. (2015) 121(3):913–22. 10.2466/10.PMS.121c23x0 26595205

[16] Delorme N Boiché J Raspaud M. Relative age effect in female sport: a diachronic examination of soccer players. Scand J Med Sci Sports. (2010) 20(3):509–15. 10.1111/j.1600-0838.2009.00979.x 19602186

[17] Delorme N Boiché J Raspaud M. Relative age and dropout in French male soccer. J Sports Sci. (2010) 28:717–22. 10.1080/02640411003663276 20480428

[18] Erlandson MC Sherar LB Mirwald RL Maffulli N Baxter-Jones ADG. Growth and maturation of adolescent female gymnasts, swimmers, and tennis players. Med Sci Sports Exerc. (2008) 40(1):34–42. 10.1249/mss.0b013e3181596678 18182934

[19] Tanner JM Whitehouse RH Takaishi M. Standards from birth to maturity for height, weight, height velocity, and weight velocity: British children, 1965. I. Arch Dis Child. (1966) 41(219):454–71. 10.1136/adc.41.219.454 5957718 PMC2019592

[20] Largo RH Gasser TH Prader A Stuetzle W Huber PJ. Analysis of the adolescent growth spurt using smoothing spline functions. Ann Hum Biol. (1978) 5(5):421–34. 10.1080/03014467800003071 727700

[21] Lance LM. Gender differences in perceived role conflict among university student-athletes. Coll Stud J. (2004) 38(2):179.

[22] Moulds K Abbott S Pion J Brophy-Williams C Heathcote M Cobley S. Sink or swim? A survival analysis of sport dropout in Australian youth swimmers. Scand J Med Sci Sports. (2020) 30(11):2222–33. 10.1111/sms.13771 32668035

[23] Vilhjalmsson R Kristjansdottir G. Gender differences in physical activity in older children and adolescents: the central role of organized sport. Soc Sci Med. (2003) 56:363–74. 10.1016/S0277-9536(02)00042-4 12473321

[24] Brustio PR Cardinale M Lupo C Boccia G. Don’t throw the baby out with the bathwater: talent in swimming sprinting events might be hidden at early age. Int J Sports Physiol Perform. (2022) 17(11):1550–7. 10.1123/ijspp.2021-0530 35894878

[25] Brustio PR Cardinale M Lupo C Varalda M De Pasquale P Boccia G. Being a top swimmer during the early career is not a prerequisite for success: a study on sprinter strokes. J Sci Med Sport. (2021) 24(12):1272–7. 10.1016/j.jsams.2021.05.015 34099366

[26] Kearney PE Hayes PR. Excelling at youth level in competitive track and field athletics is not a prerequisite for later success. J Sports Sci. (2018) 36(21):2502–9. 10.1080/02640414.2018.1465724 29667867

