# Supplementary material for: A survival analysis of dropout among French swimmers
Source: Front Sports Act Living. 2025 Mar 5;7:1509306. doi: 10.3389/fspor.2025.1509306 (PMC11919869; doi:10.3389/fspor.2025.1509306)
Supplement: Supplementary file 1 [file Table1.docx]

| **Event** | **L1 Q1** | **L1 Q2** | **L1 Q3** | **L1 Q4** | **OR Q1-Q4** | **OR S1-S2** | **L10 Q1** | **L10 Q2** | **L10 Q3** | **L10 Q4** | **OR Q1-Q4** | **OR S1-S2** |
| --- | --- | --- | --- | --- | --- | --- | --- | --- | --- | --- | --- | --- |
| **100m Breaststroke - female** | 36,79 | 30,22 | 20,42 | 12,57 | 4,05 | 4,13 | 23,01 | 22,66 | 27,05 | 27,28 | 0,8 | 0,71 |
| **100m Breaststroke - male** | 46,96 | 26,44 | 19 | 7,6 | 10,77 | 7,62 | 18,26 | 24,05 | 26,94 | 30,75 | 0,5 | 0,54 |
| **100m Backstroke - female** | 40,05 | 26,62 | 21,76 | 11,57 | 5,1 | 4 | 25,75 | 24,13 | 22,85 | 27,26 | 0,93 | 0,99 |
| **100m Backstroke - male** | 43,35 | 28,61 | 17,92 | 10,12 | 6,8 | 6,59 | 22 | 22,87 | 26,77 | 28,36 | 0,71 | 0,66 |
| **100m Freestyle - female** | 40,44 | 27,79 | 19,05 | 12,73 | 4,66 | 4,61 | 21,85 | 25,77 | 26 | 26,38 | 0,78 | 0,83 |
| **100m Freestyle - male** | 46,31 | 28,75 | 15,65 | 9,28 | 8,43 | 9,07 | 20,37 | 23,74 | 27,4 | 28,49 | 0,64 | 0,62 |
| **100m Butterfly - female** | 40,29 | 27,07 | 16,88 | 15,76 | 3,61 | 4,26 | 23,76 | 28,87 | 23,6 | 23,76 | 1 | 1,23 |
| **100m Butterfly - male** | 44,2 | 28,55 | 17,86 | 9,39 | 7,64 | 7,12 | 21,4 | 22,88 | 27,12 | 28,6 | 0,68 | 0,63 |
| **1500m Freestyle - female** | 26,32 | 36,84 | 26,32 | 10,53 | 3,04 | 2,94 | 44,44 | 33,33 | 5,56 | 16,67 | 4 | 12,25 |
| **1500m Freestyle - male** | 46,48 | 18,31 | 23,94 | 11,27 | 6,84 | 3,39 | 30,99 | 16,9 | 25,35 | 26,76 | 1,23 | 0,84 |
| **200m Breaststroke - female** | 41 | 27,59 | 17,62 | 13,79 | 4,34 | 4,77 | 21,46 | 29,89 | 27,97 | 20,69 | 1,05 | 1,11 |
| **200m Breaststroke - male** | 43,83 | 31,48 | 17,28 | 7,41 | 9,75 | 9,3 | 27,16 | 19,14 | 25,31 | 28,4 | 0,94 | 0,74 |
| **200m Backstroke - female** | 40,63 | 23,83 | 23,83 | 11,72 | 5,15 | 3,29 | 28,63 | 28,63 | 20,78 | 21,96 | 1,43 | 1,79 |
| **200m Backstroke - male** | 40,78 | 27,93 | 19,55 | 11,73 | 5,18 | 4,82 | 24,02 | 24,58 | 26,26 | 25,14 | 0,94 | 0,89 |
| **200m Freestyle - female** | 36,99 | 31,33 | 17,88 | 13,81 | 3,67 | 4,65 | 25,49 | 23,89 | 25,13 | 25,49 | 1 | 0,95 |
| **200m Freestyle - male** | 44,47 | 28,22 | 18,74 | 8,58 | 8,53 | 7,08 | 22,57 | 20,99 | 28,44 | 27,99 | 0,75 | 0,6 |
| **200m Butterfly - female** | 34,34 | 21,21 | 20,2 | 24,24 | 1,63 | 1,56 | 27,27 | 23,23 | 25,25 | 24,24 | 1,17 | 1,04 |
| **200m Butterfly - male** | 42,5 | 27,5 | 16,25 | 13,75 | 4,64 | 5,44 | 18,75 | 22,5 | 38,75 | 20 | 0,92 | 0,49 |
| **400m Freestyle - female** | 37,15 | 28,54 | 21,18 | 13,14 | 3,91 | 3,66 | 24,49 | 27,89 | 22,34 | 25,28 | 0,96 | 1,21 |
| **400m Freestyle - male** | 41,9 | 29,18 | 18,7 | 10,22 | 6,33 | 6,04 | 25,81 | 27,18 | 24,19 | 22,82 | 1,18 | 1,27 |
| **50m Breaststroke - female** | 39,81 | 27,71 | 20,17 | 12,31 | 4,71 | 4,32 | 21,55 | 25,27 | 25,16 | 28,03 | 0,71 | 0,77 |
| **50m Breaststroke - male** | 48,61 | 28,08 | 16,69 | 6,62 | 13,34 | 10,82 | 22,86 | 22,19 | 28,21 | 26,74 | 0,81 | 0,67 |
| **50m Backstroke - female** | 39,69 | 27,05 | 21,24 | 12,02 | 4,82 | 4,02 | 21,82 | 27,14 | 22,86 | 28,18 | 0,71 | 0,92 |
| **50m Backstroke - male** | 43,26 | 29,39 | 16,67 | 10,69 | 6,37 | 7,05 | 20,31 | 24,52 | 26,69 | 28,48 | 0,64 | 0,66 |
| **50m Freestyle - female** | 40,3 | 28,13 | 19,11 | 12,46 | 4,74 | 4,7 | 20,78 | 26,74 | 24,3 | 28,18 | 0,67 | 0,82 |
| **50m Freestyle - male** | 47,22 | 28,17 | 15,83 | 8,78 | 9,29 | 9,39 | 18,43 | 22,45 | 27,6 | 31,53 | 0,49 | 0,48 |
| **50m Butterfly - female** | 39,72 | 26,34 | 21,2 | 12,74 | 4,51 | 3,79 | 23,05 | 26,19 | 25,54 | 25,22 | 0,89 | 0,94 |
| **50m Butterfly - male** | 45,87 | 28,97 | 17,53 | 7,62 | 10,27 | 8,85 | 21,63 | 23,03 | 27,35 | 27,99 | 0,71 | 0,65 |
| **800m Freestyle - female** | 39,24 | 29,75 | 20,25 | 10,76 | 5,36 | 4,95 | 26,58 | 20,25 | 31,01 | 22,15 | 1,27 | 0,78 |
| **800m Freestyle - male** | 42,86 | 17,46 | 23,81 | 15,87 | 3,98 | 2,31 | 19,05 | 28,57 | 19,05 | 33,33 | 0,47 | 0,83 |

**Table 1**: Birth quarter distributions for the first quarter (Q1) and the last quarter (Q4) of the year considering performance deciles 1 (L1) and 10 (L10) at 13

| **Event** | **L1 Q1** | **L1 Q2** | **L1 Q3** | **L1 Q4** | **OR Q1-Q4** | **OR S1-S2** | **L10 Q1** | **L10 Q2** | **L10 Q3** | **L10 Q4** | **OR Q1-Q4** | **OR S1-S2** |
| --- | --- | --- | --- | --- | --- | --- | --- | --- | --- | --- | --- | --- |
| **100m Breaststroke - female** | 37,99 | 21,88 | 26,14 | 13,98 | 3,77 | 2,23 | 24,32 | 27,96 | 24,92 | 22,8 | 1,09 | 1,2 |
| **100m Breaststroke - male** | 33,33 | 28,74 | 20,05 | 17,87 | 2,3 | 2,68 | 24,15 | 27,05 | 24,88 | 23,91 | 1,01 | 1,1 |
| **100m Backstroke - female** | 29,28 | 23,57 | 24,81 | 22,33 | 1,44 | 1,26 | 22,83 | 24,57 | 25,06 | 27,54 | 0,78 | 0,81 |
| **100m Backstroke - male** | 30,56 | 31,41 | 21,37 | 16,67 | 2,2 | 2,65 | 21 | 25,76 | 22,94 | 30,3 | 0,61 | 0,77 |
| **100m Freestyle - female** | 29,35 | 28,5 | 21,68 | 20,46 | 1,62 | 1,88 | 23,39 | 28,14 | 23,87 | 24,6 | 0,94 | 1,13 |
| **100m Freestyle - male** | 33,69 | 29,28 | 22,43 | 14,59 | 2,97 | 2,89 | 21,99 | 26,15 | 25,16 | 26,7 | 0,77 | 0,86 |
| **100m Butterfly - female** | 27,91 | 25,77 | 24,23 | 22,09 | 1,37 | 1,34 | 20,99 | 23,46 | 30,56 | 25 | 0,8 | 0,64 |
| **100m Butterfly - male** | 33,93 | 29,59 | 21,89 | 14,6 | 3 | 3,03 | 22,97 | 22,97 | 28,32 | 25,74 | 0,86 | 0,72 |
| **1500m Freestyle - female** | 32,2 | 28,81 | 22,03 | 16,95 | 2,33 | 2,45 | 32,2 | 33,9 | 18,64 | 15,25 | 2,64 | 3,8 |
| **1500m Freestyle - male** | 26,88 | 23,13 | 27,5 | 22,5 | 1,27 | 1 | 22,5 | 25,63 | 24,38 | 27,5 | 0,77 | 0,86 |
| **200m Breaststroke - female** | 35,27 | 24,64 | 26,57 | 13,53 | 3,48 | 2,23 | 18,54 | 30,73 | 21,95 | 28,78 | 0,56 | 0,94 |
| **200m Breaststroke - male** | 34,8 | 28,8 | 20,4 | 16 | 2,8 | 3,05 | 24,5 | 25,7 | 25,3 | 24,5 | 1 | 1,02 |
| **200m Backstroke - female** | 27,6 | 29,2 | 23,2 | 20 | 1,52 | 1,73 | 16,8 | 32,4 | 26,4 | 24,4 | 0,63 | 0,94 |
| **200m Backstroke - male** | 26,69 | 30,74 | 21,96 | 20,61 | 1,4 | 1,82 | 23,65 | 30,74 | 22,3 | 23,31 | 1,02 | 1,42 |
| **200m Freestyle - female** | 27,32 | 30 | 21,25 | 21,43 | 1,38 | 1,8 | 20,04 | 27,37 | 26,83 | 25,76 | 0,72 | 0,81 |
| **200m Freestyle - male** | 33,01 | 28,04 | 24,86 | 14,09 | 3,01 | 2,46 | 22,13 | 26 | 26 | 25,86 | 0,81 | 0,86 |
| **200m Butterfly - female** | 27,2 | 29,6 | 27,2 | 16 | 1,96 | 1,73 | 26,61 | 29,84 | 25,81 | 17,74 | 1,68 | 1,68 |
| **200m Butterfly - male** | 31,34 | 22,39 | 30,85 | 15,42 | 2,5 | 1,35 | 24,88 | 25,37 | 30,35 | 19,4 | 1,38 | 1,02 |
| **400m Freestyle - female** | 27,62 | 32,04 | 21,55 | 18,78 | 1,65 | 2,19 | 19,89 | 26,24 | 29,83 | 24,03 | 0,78 | 0,73 |
| **400m Freestyle - male** | 30,92 | 27,63 | 23,46 | 17,98 | 2,04 | 2 | 23,25 | 24,12 | 27,41 | 25,22 | 0,9 | 0,81 |
| **50m Breaststroke - female** | 35,93 | 23,96 | 23,4 | 16,71 | 2,8 | 2,23 | 22,56 | 30,92 | 21,73 | 24,79 | 0,88 | 1,32 |
| **50m Breaststroke - male** | 34,12 | 26,23 | 21,32 | 18,34 | 2,31 | 2,31 | 22,01 | 24,57 | 28,42 | 25 | 0,85 | 0,76 |
| **50m Backstroke - female** | 33,11 | 22,82 | 20,81 | 23,27 | 1,63 | 1,61 | 21,35 | 26,97 | 23,37 | 28,31 | 0,69 | 0,87 |
| **50m Backstroke - male** | 33,92 | 28,04 | 22,16 | 15,88 | 2,72 | 2,65 | 19,45 | 30,06 | 24,95 | 25,54 | 0,7 | 0,96 |
| **50m Freestyle - female** | 29,57 | 27,28 | 22,72 | 20,43 | 1,63 | 1,74 | 22,27 | 29,74 | 24,34 | 23,65 | 0,93 | 1,17 |
| **50m Freestyle - male** | 34,01 | 30,91 | 21,08 | 13,99 | 3,17 | 3,43 | 20,96 | 26,29 | 26,02 | 26,74 | 0,73 | 0,8 |
| **50m Butterfly - female** | 30,13 | 26,32 | 24,32 | 19,24 | 1,81 | 1,68 | 25,45 | 25,45 | 23,27 | 25,82 | 0,98 | 1,08 |
| **50m Butterfly - male** | 31,73 | 30,66 | 23,69 | 13,92 | 2,87 | 2,75 | 20,94 | 26,44 | 26,71 | 25,91 | 0,76 | 0,81 |
| **800m Freestyle - female** | 32 | 29,71 | 20,57 | 17,71 | 2,19 | 2,6 | 20 | 32 | 25,14 | 22,86 | 0,84 | 1,17 |
| **800m Freestyle - male** | 27,56 | 24,36 | 28,21 | 19,87 | 1,53 | 1,17 | 16,67 | 31,41 | 26,28 | 25,64 | 0,58 | 0,86 |

**Table 2**: Birth quarter distributions for the first quarter (Q1) and the last quarter (Q4) of the year considering performance deciles 1 (L1) and 10 (L10) at 17
