# Supplementary material for: A survival analysis of dropout among French swimmers
Source: Front Sports Act Living. 2025 Mar 5;7:1509306. doi: 10.3389/fspor.2025.1509306 (PMC11919869; doi:10.3389/fspor.2025.1509306)
Supplement: Supplementary file 2 [file Table2.docx]

| **Event** | **Age** | **L1 (%)** | **L10 (%)** | **Age** | **L1 (%)** | **L10 (%)** |
| --- | --- | --- | --- | --- | --- | --- |
| **50m Freestyle - male** | 13 | 9,9 | 69,3 | 17 | 28,6 | 83,4 |
| **50m Freestyle - female** | 13 | 8,7 | 78,1 | 17 | 39,6 | 91,7 |
| **100m Freestyle - male** | 13 | 6,6 | 71,7 | 17 | 27,2 | 84,7 |
| **100m Freestyle - female** | 13 | 9,3 | 78,8 | 17 | 38,3 | 91 |
| **200m Freestyle - male** | 13 | 5,8 | 68,6 | 17 | 28,2 | 91,6 |
| **200m Freestyle - female** | 13 | 6,7 | 80,5 | 17 | 41,4 | 93 |
| **400m Freestyle - male** | 13 | 13,1 | 81,2 | 17 | 30,7 | 91,4 |
| **400m Freestyle - female** | 13 | 14,4 | 85,2 | 17 | 43,1 | 96,9 |
| **800m Freestyle - male** | 13 | 23,8 | 88,9 | 17 | 37,8 | 95,8 |
| **800m Freestyle - female** | 13 | 10 | 78,5 | 17 | 48,6 | 97,1 |
| **1500m Freestyle - male** | 13 | 17,6 | 81,8 | 17 | 37,9 | 93,9 |
| **1500m Freestyle - female** | 13 | 50 | 100 | 17 | 43,8 | 100 |
| **50m Backstroke - male** | 13 | 14,4 | 84,2 | 17 | 32,9 | 90,1 |
| **50m Backstroke - female** | 13 | 16,2 | 86,5 | 17 | 41,5 | 93,4 |
| **100m Backstroke - male** | 13 | 11,4 | 80,5 | 17 | 29,2 | 92,4 |
| **100m Backstroke - female** | 13 | 9 | 84,3 | 17 | 38,4 | 95,4 |
| **200m Backstroke - male** | 13 | 6 | 77,9 | 17 | 28 | 93,3 |
| **200m Backstroke - female** | 13 | 2,9 | 78,2 | 17 | 38,6 | 95,6 |
| **50m Breaststroke - male** | 13 | 19,6 | 84,7 | 17 | 34,8 | 92,1 |
| **50m Breaststroke - female** | 13 | 20,6 | 89,2 | 17 | 47,2 | 92,7 |
| **100m Breaststroke - male** | 13 | 14,7 | 88,9 | 17 | 28,6 | 92,4 |
| **100m Breaststroke - female** | 13 | 13 | 88,5 | 17 | 36 | 94,1 |
| **200m Breaststroke - male** | 13 | 13,6 | 84,1 | 17 | 26,6 | 92,9 |
| **200m Breaststroke - female** | 13 | 12,2 | 86,8 | 17 | 32,9 | 94,9 |
| **50m Butterfly - male** | 13 | 7,8 | 75,7 | 17 | 31,2 | 85,9 |
| **50m Butterfly - female** | 13 | 7,9 | 79 | 17 | 38,8 | 91,9 |
| **100m Butterfly - male** | 13 | 4 | 73,2 | 17 | 28,6 | 88,5 |
| **100m Butterfly - female** | 13 | 10,6 | 88,6 | 17 | 34,9 | 91,2 |
| **200m Butterfly - male** | 13 | 7,4 | 73 | 17 | 24,5 | 89,9 |
| **200m Butterfly - female** | 13 | 0 | 29,6 | 17 | 37 | 95 |

**Table 3**: Dropout rates at age 14 according to performance deciles 1 (L1) and 10 (L10) at 13 years old and at age 18 according to performance deciles 1 and 10 at 17 years old.
